# Annexin A1 restores Aβ_1‐42_‐induced blood–brain barrier disruption through the inhibition of RhoA‐ROCK signaling pathway

**DOI:** 10.1111/acel.12530

**Published:** 2016-09-16

**Authors:** Jong‐Chan Park, Sung Hoon Baik, Sun‐Ho Han, Hyun Jin Cho, Hyunjung Choi, Haeng Jun Kim, Heesun Choi, Wonik Lee, Dong Kyu Kim, Inhee Mook‐Jung

**Affiliations:** ^1^ Department of Biochemistry and Biomedical SciencesCollege of MedicineSeoul National UniversitySeoul110‐799Korea

**Keywords:** 5XFAD mice, Alzheimer's disease, Annexin A1, blood–brain barrier, endothelial cell, pericyte, β‐amyloid

## Abstract

The blood–brain barrier (BBB) is composed of brain capillary endothelial cells and has an important role in maintaining homeostasis of the brain separating the blood from the parenchyma of the central nervous system (CNS). It is widely known that disruption of the BBB occurs in various neurodegenerative diseases, including Alzheimer's disease (AD). Annexin A1 (ANXA1), an anti‐inflammatory messenger, is expressed in brain endothelial cells and regulates the BBB integrity. However, its role and mechanism for protecting BBB in AD have not been identified. We found that β‐Amyloid 1‐42 (Aβ42)‐induced BBB disruption was rescued by human recombinant ANXA1 (hrANXA1) in the murine brain endothelial cell line bEnd.3. Also, ANXA1 was decreased in the bEnd.3 cells, the capillaries of 5XFAD mice, and the human serum of patients with AD. To find out the mechanism by which ANXA1 recovers the BBB integrity in AD, the RhoA‐ROCK signaling pathway was examined in both Aβ42‐treated bEnd.3 cells and the capillaries of 5XFAD mice as RhoA was activated in both cases. RhoA inhibitors alleviated Aβ42‐induced BBB disruption and constitutively overexpressed RhoA‐GTP (active form of RhoA) attenuated the protective effect of ANXA1. When pericytes were cocultured with bEnd.3 cells, Aβ42‐induced RhoA activation of bEnd.3 cells was inhibited by the secretion of ANXA1 from pericytes. Taken together, our results suggest that ANXA1 restores Aβ42‐induced BBB disruption through inhibition of RhoA‐ROCK signaling pathway and we propose ANXA1 as a therapeutic reagent, protecting against the breakdown of the BBB in AD.

## Introduction

The blood–brain barrier (BBB) is comprised of vascular endothelial cells, which are connected by tight junctions with a highly selective permeability by way of active transport mechanism (Pardridge, 2012). Although it has extremely high electrical resistivity and compact tight junctions, the BBB allows gases, water, small lipid soluble compounds, and hydrophobic molecules by passive diffusion as well as selective transport of molecules that are highly crucial to the neurological function such as amino acids and glucose (Butt *et al*., 1990; Hawkins *et al*., 2002). The BBB blocks the entry of harmful substances such as neurotoxins, bacteria, and other detrimental chemicals, in contrast to other peripheral organs which can have leaky blood vessels (Mann *et al*., 1985). It has been reported that the BBB maintains their integrity by the help of other cells, and interaction between pericytes and endothelial cells is a vital requisite for the BBB maintenance (Zhao *et al*., 2015). For example, endothelial cells secrete platelet‐derived growth factor‐β (PDGFβ) to recruit pericytes to survive against abnormal tumor vasculature (Chatterjee & Naik, 2012), and pericytes regulate endothelial cells by releasing cytokines (LaBarbera *et al*., 2015). In this way, the BBB has a critical role in maintaining homeostasis of the brain and separates the blood from the parenchyma of the central nervous system (CNS), as the major regulator between the brain and remainder of the body (Ballabh *et al*., 2004; Cristante *et al*., 2013) with the support of pericytes, which are generally known as key cells of the BBB maintenance.

It is well known that the integrity of the BBB is damaged in many neurodegenerative diseases such as Alzheimer's disease (AD), vascular dementia, multiple sclerosis (MS), and Parkinson's disease (PD) (Cristante *et al*., 2013; Erickson & Banks, 2013; Gray & Woulfe, 2015). Particularly in AD, there are some alterations of the cerebral vascular structure that lead to cerebral amyloid angiopathy (CAA), decrease in tight junction proteins, and elevation of the BBB permeability during the progress of disease (Kook *et al*., 2012). Also, it has been reported that β‐Amyloid 1‐42 (Aβ42) induced an increase in the BBB permeability and disruption of tight junction proteins through several mechanisms, such as oxidative stress associated pathway, up‐regulation of receptor for advanced glycation end products (RAGEs), and enhanced expression of matrix metalloproteinase 9 (Barnham *et al*., 2004; Kook *et al*., 2012). However, there are still many unknown mechanisms and dissenting opinions (Bien‐Ly *et al*., 2015) about the BBB dysfunction in the mouse model and in patients with AD.

Annexin A1 (ANXA1), one of the anti‐inflammatory factors, has neuroprotective effects by the removal of apoptotic cells (McArthur *et al*., 2010), the protecting neurons from ischemia‐like injury (Luo *et al*., 2014), and the prevention of hepatic inflammation (Locatelli *et al*., 2014). In particular, ANXA1 can attenuate the breakdown of the BBB, and its expression level was reduced in the brain capillaries of patients with MS (Cristante *et al*., 2013). However, exact role and mechanism of ANXA1 for protecting the BBB in AD have not been reported. We hypothesized that ANXA1 protects the BBB in AD and tried to find out the mechanism. Utilizing the murine brain endothelial cell line (bEnd.3) and the 5XFAD transgenic AD mouse model, we have shown that Aβ42‐induced BBB disruption is mediated by the RhoA‐ROCK signaling pathway and showed that ANXA1 can alleviate the BBB breakdown through inhibition of the RhoA‐ROCK signaling pathway. When pericytes were cocultured with bEnd.3 cells, Aβ‐induced RhoA activation of bEnd.3 cells was inhibited by secretion of ANXA1 from pericytes. Finally, we identified a down‐regulation of ANXA1 expression in the serum of patients with AD, suggesting an accelerated BBB breakdown due to the lack of protective role by ANXA1 in AD. Our results demonstrate the critical roles of ANXA1 in the maintenance of the BBB integrity and suggest a possible therapeutic target for the protection of the BBB in AD.

## Results

### BBB is disrupted in the Aβ42‐treated bEnd.3 cells and 5XFAD mice

**Figure 1:**
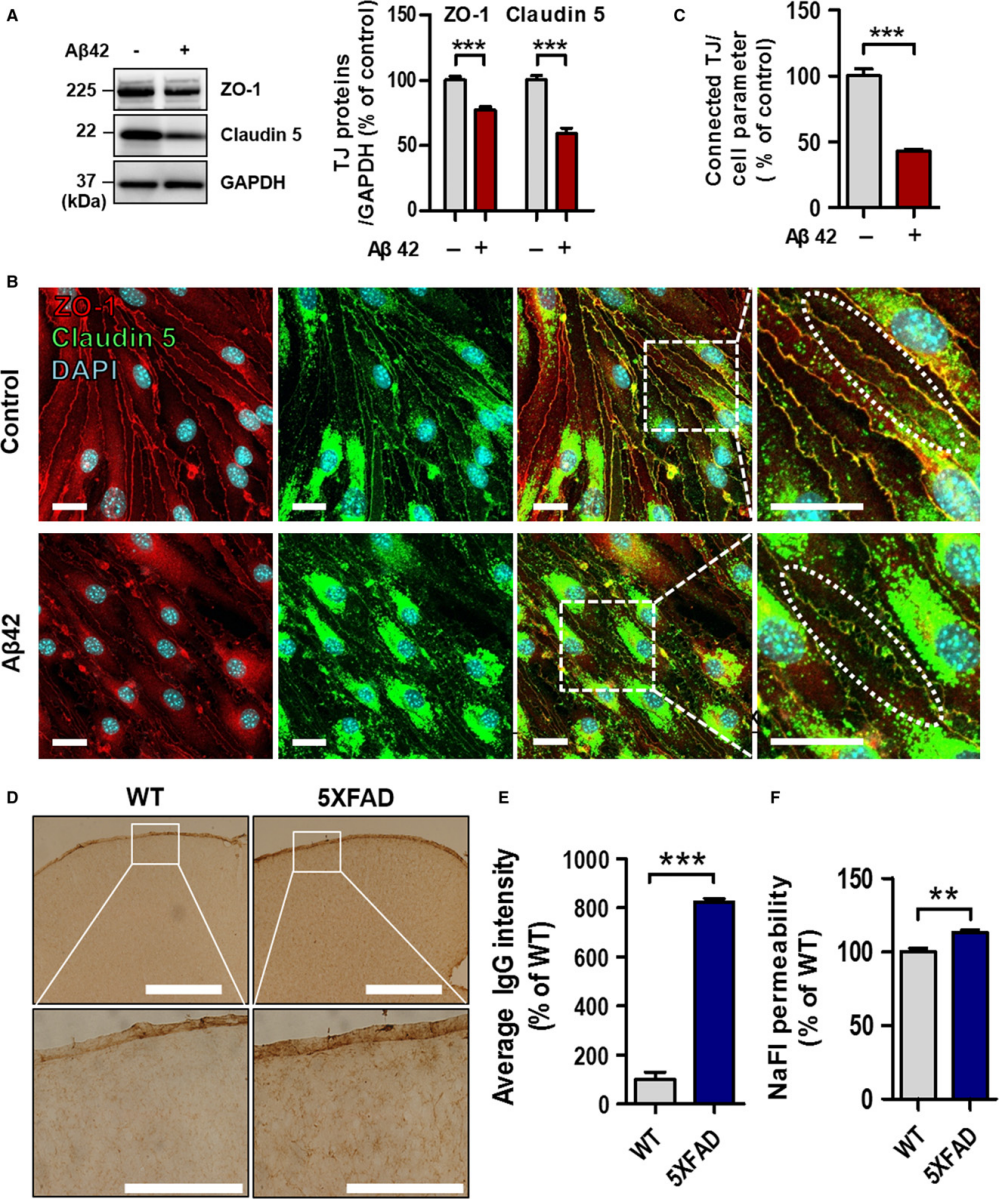
Aβ42 induces blood–brain barrier (BBB) disruption. (A) Levels of ZO‐1 and Claudin 5 are decreased in Aβ42‐treated bEnd.3 cells. GAPDH was used as a loading control (Aβ42, 5 μm for 24 h; *n* = 5 independent experiments; ***P < 0.001 vs. vehicle, unpaired *t*‐test). (B) Representative confocal microscopy images of ZO‐1 and Claudin 5 in a monolayer culture of bEnd.3 cells. Disconnected short length of tight junction and nonoverlapping ZO‐1 and Claudin 5 signal (elliptic dotted line) were observed in the Aβ42 treated bEnd.3 cells. Scale bar, 20 μm; elliptic dotted line indicates connected (control) or disconnected (Aβ42) tight junctions. Yellow signals show the colocalization of ZO‐1 and Claudin 5. (C) Quantification of connected tight junction length by Image J software, divided by average total length of cell borders (***P < 0.001 vs. control, unpaired *t*‐test). (D,E) Representative images of IgG staining. Scale bar, 50 μm (upper row) and 200 μm (lower row) (***P < 0.001 vs. WT, unpaired *t*‐test). (F) *In vivo* BBB permeability in wild‐type and 5XFAD mice (each, *n* = 4); 0.1 mL of sodium fluorescein (NaFI, 0.1 g mL^−1^; 0.376 kDa) was intraperitoneally administered (**P < 0.01 vs. WT, unpaired *t*‐test). TJ, tight junction; NaFI, sodium fluorescein.

To measure the BBB disruption *in vitro* and *in vivo*, we examined the levels of the tight junction proteins in bEnd.3 cells and performed IgG staining and an *in vivo* BBB permeability assay using sodium fluorescein (NaFI). After treatment of Aβ42 on a monolayer of bEnd.3 cells, Western blotting showed that the levels of ZO‐1 and Claudin 5 were significantly decreased (Fig. 1A, ***P < 0.001) as we reported previously (Kook *et al*., 2012). Immunocytochemistry with antibodies against ZO‐1 and Claudin 5 showed fragmented patterns and less colocalization of both proteins in Aβ42‐treated bEnd.3 cells compared to the control (Fig. 1B, elliptic dotted line showed tight junctions and yellow signals showed the colocalization of ZO‐1 and Claudin 5). When the length of tight junction was measured, it was significantly decreased in Aβ42‐treated bEnd.3 cells compared to the control (Fig. 1C, ***P < 0.001), implying that Aβ42 induces disruption of the tight junction proteins of bEnd.3 cells. To examine whether the disruption of tight junction proteins is due to the cell death, cell viability assay was performed under condition of Aβ42 treatment on bEnd.3 cells. MTT assay showed no significant difference between two groups (Fig. S2, *P* > 0.05). To examine the leakage of the BBB *in vivo*, IgG extravasation was tested with the immunohistochemistry followed by intensity measurement in the brain of 9‐ to 10‐month‐old 5XFAD mice (Fig. 1D,E, ***P < 0.001). Also, sodium fluorescein (NaFI) permeability assay was performed in 5XFAD mice (Fig. 1F, **P < 0.01). Both assays showed that the permeability was significantly increased in 5XFAD mice compared to the wild‐type mice, implying that the BBB breakdown was existed in the brains of 5XFAD mice.

### ANXA1 is decreased in the serum of patients with AD

**Figure 2:**
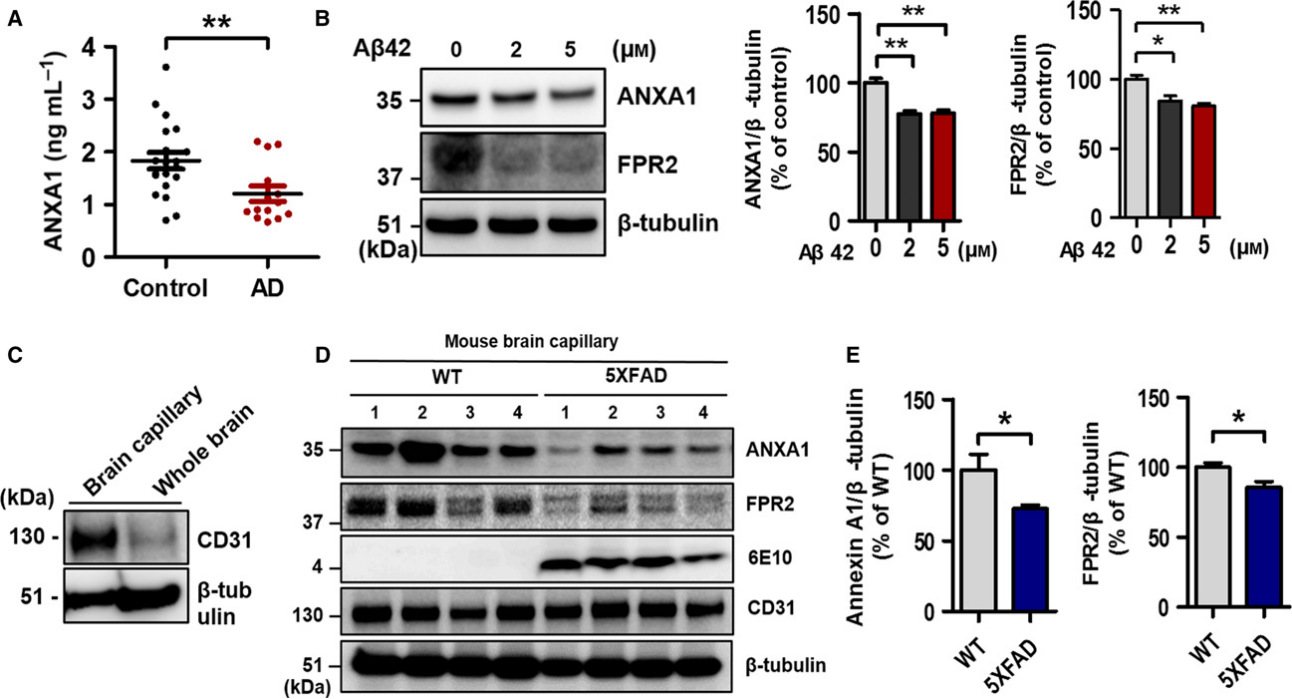
The levels of ANXA1 and FPR2 in the condition of AD. (A) Quantification of the levels of ANXA1 in human serum by ELISA (Controls, *n* = 20; AD,* n* = 14; **P < 0.01, AD vs. control group, unpaired *t*‐test). (B) ANXA1 and FPR2 were significantly reduced by the treatment of Aβ42 (0, 2, and 5 μm for 24 h) in the bEnd.3 cells (*n* = 3 independent experiments; *P < 0.05 and **P < 0.01, ANOVA with Tukey's multiple‐comparison test). (C) Experimental validation of mouse brain capillary isolation (CD31, endothelial cell marker; β‐tubulin, loading control). (D–E) ANXA1 and FPR2 were decreased in the capillary of 5XFAD mice (each, *n* = 8; ANXA1, *P < 0.05 vs. wild‐type mice; FPR2, *P < 0.05 vs. wild‐type mice; unpaired *t*‐test). Anti‐CD31 antibody was used to confirm the isolation of mouse brain capillaries, β‐tubulin was used as a loading control, and anti‐6E10 antibody was used to check Aβ42 in the 5XFAD transgenic mice.

As previous studies show that exogenous ANXA1 has neuroprotective effects and helps in maintaining the BBB integrity (Cristante *et al*., 2013), it seemed reasonable to check the level of ANXA1 in of the blood samples of patients with AD. We measured ANXA1 level in the serum of the subjects (Controls, *n* = 20; patients with AD, *n* = 14) by enzyme‐linked immunosorbent assay (ELISA) and showed that it was significantly reduced in the serum of patients with AD (Fig. 2A, **P < 0.01). This suggests that the reduced level of ANXA1 in the serum induces BBB dysfunction in AD.

### ANXA1 and FPR2 are decreased in the Aβ42‐treated bEnd.3 cells and the capillary of 5XFAD mice

Next we examined the possible contribution of the endogenous ANXA1 and its receptor FPR2 on the BBB disruption in AD utilizing Aβ42‐treated bEnd.3 cells and the brain capillaries of 9‐ to 10‐month‐old 5XFAD mice. Both ANXA1 and FPR2 expressions were significantly reduced by the treatment of Aβ42 for 24 h in bEnd.3 cells (Fig. 2B, *P < 0.05 and **P < 0.01). In addition, the levels of both ANXA1 and FPR2 were reduced in the brain capillaries of 5XFAD mice (Fig. 2D,E; ANXA1, *P < 0.05; FPR2, *P < 0.05).

### BBB impairment is attenuated by human recombinant ANXA1 in the Aβ42‐treated bEnd.3 cells

**Figure 3:**
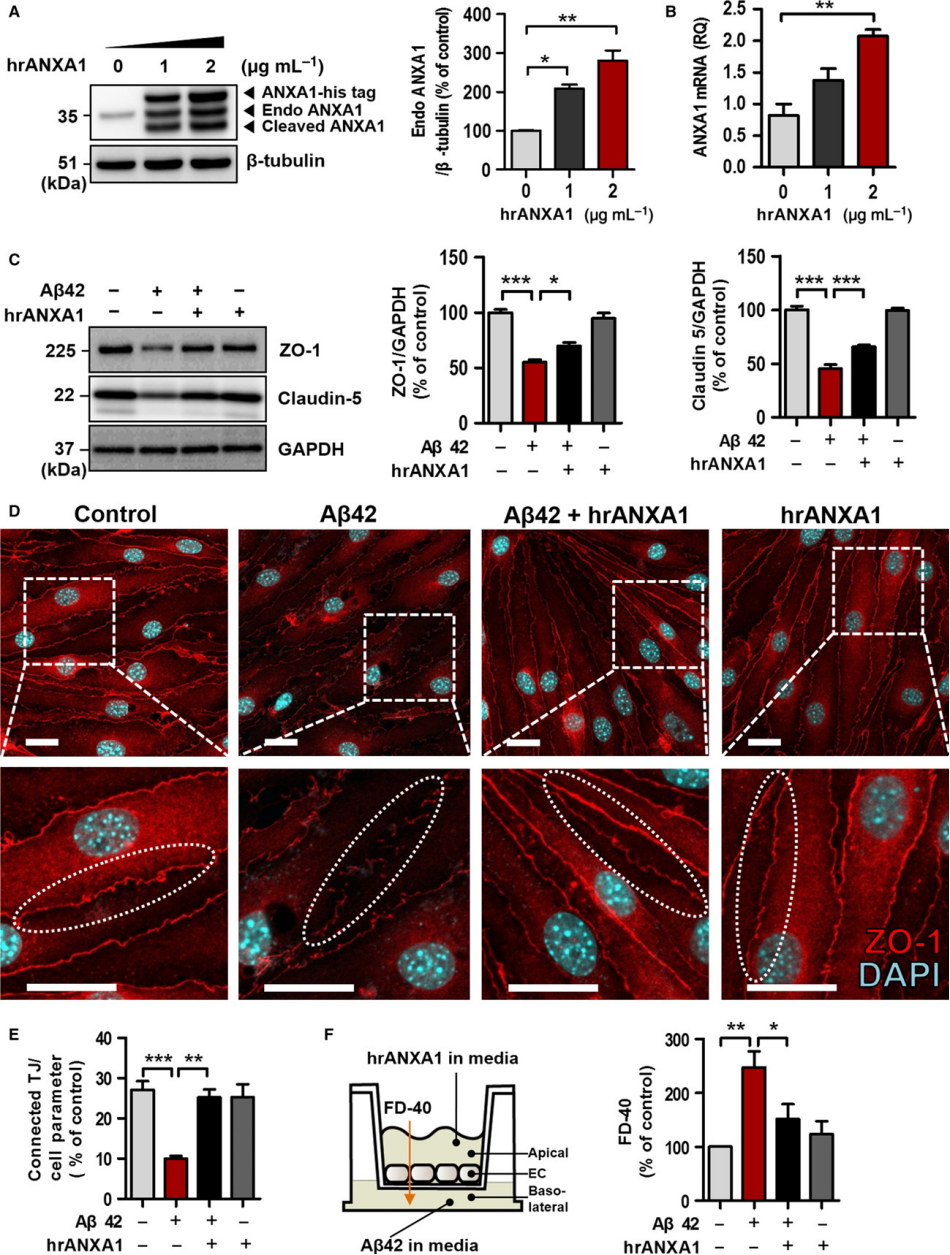
Exogenous hrANXA1 restores the blood–brain barrier (BBB) disruption in Aβ42‐treated bEnd.3 cells. (A) Endogenous ANXA1 was increased dose dependently by (0, 1, and 2 μg mL^−1^ for 24 h) exogenously treated hrANXA1 protein in the bEnd.3 cells (35.5 kDa; *P < 0.05 and **P < 0.01, *n* = 2, ANOVA with Tukey's multiple‐comparison test). After treatment of hrANXA1 protein (His‐tagged form, 42 kDa), his‐tagged hrANXA1 proteins (42 kDa) and internalized cleaved ANXA1 (33 kDa) were also captured by anti‐ANXA1 antibody. β‐Tubulin was used as a loading control. (B) The level of mRNA was increased in the bEnd.3 cells by treatment of hrANXA1 (0, 1, and 2 μg mL^−1^ for 24 h; **P < 0.01; each, *n* = 3, ANOVA with Tukey's multiple‐comparison test). (C) bEnd.3 cells were pretreated with hrANXA1 (1 μg mL^−1^ for 30 min) and then incubated with Aβ42 (5 μm for 24 h). *n* = 5 independent experiments (*P < 0.05 and ***P < 0.001, ANOVA with Tukey's multiple‐comparison test). (D–E) Confocal microscopic image of ZO‐1 (red) proteins and quantification of connected tight junction (TJ) length in bEnd.3 cells. Connected TJ length was measured by Image J software and divided by total length of cell borders. Elliptic dotted line indicates connected or disconnected tight junctions. See Experimental procedure section (Aβ42, 5 μm for 24 h; hrANXA1, 5 μg mL^−1^, pretreated for 30 min before Aβ42 treatment and incubated with Aβ42 for 24 h; each, *n* = 4 independent experiments; **P < 0.01 and ***P < 0.001, ANOVA with Tukey's multiple‐comparison test; Scale bar, 20 μm). (F) *In vitro* transwell BBB permeability assay. hrANXA1 was pretreated in the apical side of transwell (1 μg mL^−1^, 30 min before Aβ42 treatment), and then Aβ42, was also treated in the basolateral side of transwell (5 μm, 24 h). FITC‐dextran 40 (40 kDa, 0.1 mg mL^−1^ for 30 min; FD‐40) was used to measure the permeability of bEnd.3 cells (*P < 0.05 and **P < 0.01, ANOVA with Tukey's multiple‐comparison test; each, *n* = 6). TJ, tight junction; hrANXA1, human recombinant protein Annexin A1.

Because ANXA1 was reduced in the Aβ42‐treated bEnd.3 cells and capillaries of 5XFAD mice, it is reasonable to examine whether the treatment of human recombinant protein ANXA1 (hrANXA1) affects endogenous ANXA1 levels and increases the BBB integrity and tight junction protein levels. The treatment of hrANXA1 (His‐tagged form, 42 kDa) increased the level of endogenous ANXA1 protein in a dose‐dependent manner (35.5 kDa; *P < 0.05 and **P < 0.01, Fig. 3A) in the bEnd.3 cells. His‐tagged hrANXA1 (42 kDa) and internalized cleaved hrANXA1 (33 kDa) (Perretti & Flower, 2004; Vong *et al*., 2012) were also detected by anti‐ANXA1 antibody. Moreover, elevated ANXA1 mRNA level (Fig. 3B, **P < 0.01) by the treatment of hrANXA1 clearly showed that exogenously treated hrANXA1 directly increased endogenous ANXA1 expression level. Next, to show that hrANXA1 increases the BBB integrity in the bEnd.3 cells, the cells were treated with hrANXA1 followed by the treatment of Aβ42 (hrANXA1, 1 μg mL^−1^ for 30 min pretreatment followed by incubation with 5 μm Aβ42 for 24 h). Consequently, the expression of reduced tight junction proteins by Aβ42 treatment was significantly rescued by hrANXA1 protein (Fig. 3C; ZO‐1, *P < 0.05; Claudin 5, ***P < 0.001). Further immunofluorescence analysis by the confocal microscopy showed the recovery of connected tight junction length by the treatment of hrANXA1 (Fig. 3D,E, **P < 0.01 and ***P < 0.001; elliptic dotted line, tight junction). Also, the result of *in vitro* BBB permeability assay using FD‐40 (fluorescein isothiocyanate dextran, 40 kDa) showed that the pretreatment of hrANXA1 (in apical chamber of transwell insert) for 30 min significantly reduced membrane permeability increased by Aβ42 (in basolateral chamber of transwell) (Fig. 3F, *P < 0.05 and **P < 0.01). Therefore, these data showed that ANXA1 restores Aβ42‐induced BBB disruption in the bEnd.3 cells.

### RhoA‐ROCK signaling pathway is activated in the Aβ42‐treated bEnd.3 cells and the capillary of 5XFAD mice

**Figure 4:**
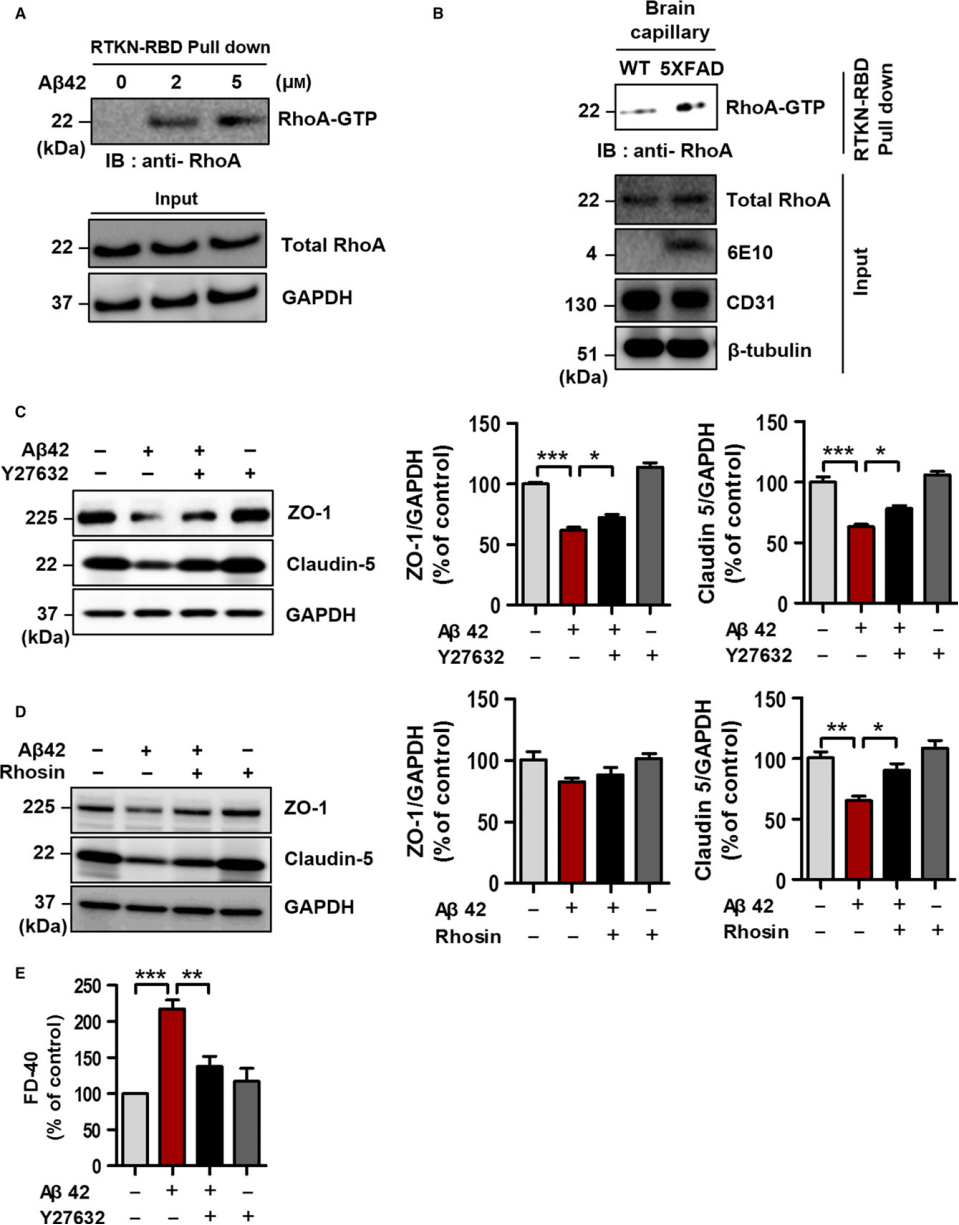
RhoA is activated in Aβ42‐treated bEnd.3 cells and the capillary of 5XFAD mice, and inhibition of RhoA‐ROCK signaling pathway restores the blood–brain barrier (BBB) disruption. RhoA‐GTP was measured by RhoA‐GTP pull‐down assay. Rhotekin‐RBD beads were used to pull down the GTP‐bound form of Rho, and RhoA‐GTP was captured by anti‐RhoA antibody. (A) The level of RhoA‐GTP was measured in various doses (0, 2, and 5 μm, 30 min) with Aβ42 in bEnd.3 cells. GAPDH is used as a loading control. (B) RhoA was activated in the capillary of 5XFAD mice. Anti‐CD31 antibody was used to validate the isolation of mouse brain capillaries, β‐tubulin was used as a loading control, and anti‐6E10 antibody was used to confirm the level of Aβ in the 5XFAD transgenic mice. (C) Representative Western blot images showed the effect of Y27632 (ROCK inhibitor; 30 μm for 24 h) in the Aβ42‐treated bEnd.3 cells (5 μm for 24 h). Diminished tight junction proteins (ZO‐1 and Claudin 5) were significantly rescued by the treatment of Y27632 (30 μm for 24 h) in the Aβ42‐treated bEnd.3 cells (5 μm for 24 h) (*P < 0.05 and ***P < 0.001, ANOVA with Tukey's multiple‐comparison test; each, *n* = 5). (D) The ability of Rhosin to restore tight junction proteins (inhibitor of GEF‐catalyzed RhoA activation; 10 μm for 24 h with 5 μm Aβ42; *P < 0.05 and **P < 0.01, ANOVA with Tukey's multiple‐comparison test; each, *n* = 4). (E) *In vitro* transwell BBB permeability assay. FITC‐dextran 40 (0.1 mg mL^−1^, 40 kD; FD‐40) was used to measure the permeability of bEnd.3 cells (**P < 0.01 and ***P < 0.001, ANOVA with Tukey's multiple‐comparison test; each, *n* = 4).

It has been reported that an antisense of sequence to hrANXA1 increases RhoA‐GTP activity, but the treatment of exogenous hrANXA1 can reverse this effect, in the immortalized human brain microvascular endothelial cell (hCMEC/D3) (Cristante *et al*., 2013). We hypothesized that the protective effect of ANXA1 in AD (Fig. 3) is related to the inhibition of RhoA‐GTP. Therefore, we investigated whether the activity of RhoA is increased in the Aβ42 treated bEnd.3 cells and the capillary of 5XFAD mice using the active RhoA pull‐down assay. When Aβ42 was treated, RhoA‐GTP was dramatically increased in bEnd.3 cells (Fig. 4A). Moreover, the level of RhoA‐GTP was also enhanced in the brain capillaries of 5XFAD mice (Fig. 4B). Collectively, these data suggest that Aβ42 induces the activation of the RhoA signaling pathway and present the possibility of a correlation between RhoA activation and the BBB disruption in AD.

### Inhibition of RhoA‐ROCK signaling pathway attenuates Aβ42‐induced BBB disruption

To evaluate whether RhoA‐GTP signaling pathway mediated the Aβ42‐induced BBB disruption, we treated bEnd.3 cells with ROCK inhibitor, Y27632 (Ishizaki *et al*., 2000), or Rho‐GTPase activation site inhibitor, Rhosin (Shang *et al*., 2012, 2013) along with Aβ42. In the presence of Y27632, the reduced levels of tight junction proteins (ZO‐1 and Claudin 5) by Aβ42 were significantly rescued (Fig. 4C, *P < 0.05), and the effect of Rhosin on bEnd.3 cells was similar to that of Y27632 (Fig. 4D, Claudin 5, *P < 0.05). In addition, *in vitro* BBB permeability assay using FD‐40 showed that cotreatment of Y27632 with Aβ42 significantly reduced membrane permeability, which was enhanced by Aβ42 (Fig. 4E, **P < 0.01 and ***P < 0.001). These data indicate that Aβ42‐induced BBB disruption and the reduction in tight junction proteins are mediated by the RhoA‐ROCK signaling pathway.

### ANXA1 attenuates Aβ42‐induced tight junction dysfunction by inhibition of the RhoA‐ROCK signaling pathway

**Figure 5:**
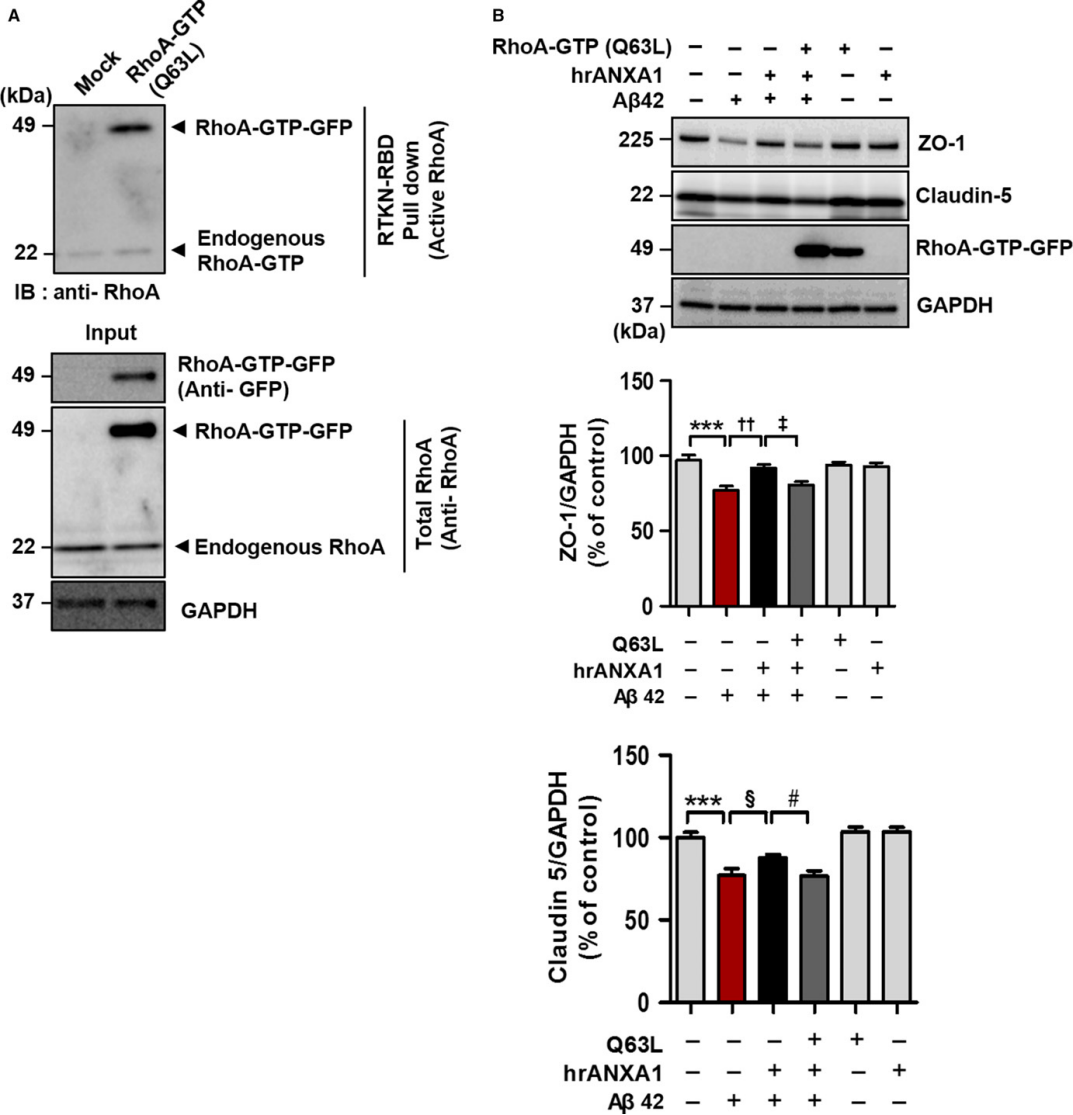
ANXA1 attenuates Aβ42‐induced blood–brain barrier (BBB) disruption by inhibition of the RhoA‐ROCK signaling pathway. RhoA‐GTP was detected by RhoA‐GTP pull‐down assay. GTP‐bound form of RhoA was pulled down by Rhotekin‐RBD beads, and RhoA‐GTP was captured by anti‐RhoA antibody. (A) Validating a mutant of RhoA‐GTP (constitutively active form, pcDNA3‐EGFP‐RhoA‐Q63L). Increased RhoA‐GTP‐GFP expression after constitutively active RhoA (GFP‐tagged) cDNA transfection was detected by both RhoA‐GTP pull‐down assay (upper blot, 49 kDa) and anti‐GFP antibody (lower blot, 49 kDa). RhoA‐GTP‐GFP was captured on 49 kDa (RhoA, 22 kDa; GFP, 27 kDa) and GAPDH was used as a loading control. (B) The protective effect of ANXA1 is inhibited by the overexpression of constitutive RhoA‐GTP. Twenty‐four hour after RhoA‐GTP transfection, bEnd.3 cells were pretreated with hrANXA1 (1 μg mL^−1^ for 30 min) and incubated with Aβ42 (5 μm for 24 h). Anti‐GFP antibody was used to validate overexpression of RhoA‐GTP‐GFP (49 kDa). Rescued ZO‐1 and Claudin 5 protein with hrANXA1 treatment (ZO‐1, ^††^ *P* < 0.01, ANOVA with Tukey's multiple‐comparison test; Claudin 5, ^§^ *P* < 0.05, unpaired *t*‐test; each *n* = 8) were significantly decreased again by RhoA activation (ZO‐1, ^‡^ *P* < 0.05, ANOVA with Tukey's multiple‐comparison test; Claudin 5, ^#^ *P* < 0.05, unpaired *t*‐test; each *n* = 8).

As Aβ42‐induced BBB disruption was attenuated by both the RhoA‐ROCK pathway inhibitors and ANXA1 protein (Figs 3 and 4), we investigated whether the protective effect of ANXA1 is related to the inhibition of RhoA‐GTP. RhoA‐GTP Q63L mutant is constitutively activated GTP‐bound form, and it was transiently transfected to bEnd.3 cells (Fig. 5A). Aβ42‐treated bEnd.3 cells without mutant of RhoA‐GTP showed significant recovery of tight junction proteins (ZO‐1 and Claudin 5) by hrANXA1 (Fig. 5B; ZO‐1, ^††^ *P* < 0.01; Claudin 5, ^§^ *P* < 0.05; each *n* = 8). However, the restored tight junction proteins by hrANXA1 were decreased again through the transfection of mutant RhoA‐GTP (Fig. 5B; ZO‐1, ^‡^ *P* < 0.05; Claudin 5, ^#^ *P* < 0.05; each *n* = 8). These results show that the constitutive activation of RhoA‐GTP inhibits the protective effect of ANXA1 against Aβ42 and suggest that ANXA1 restore BBB disruption by the inhibition of RhoA‐GTP signaling.

### Pericytes regulate BBB integrity through ANXA1 secretion

**Figure 6:**
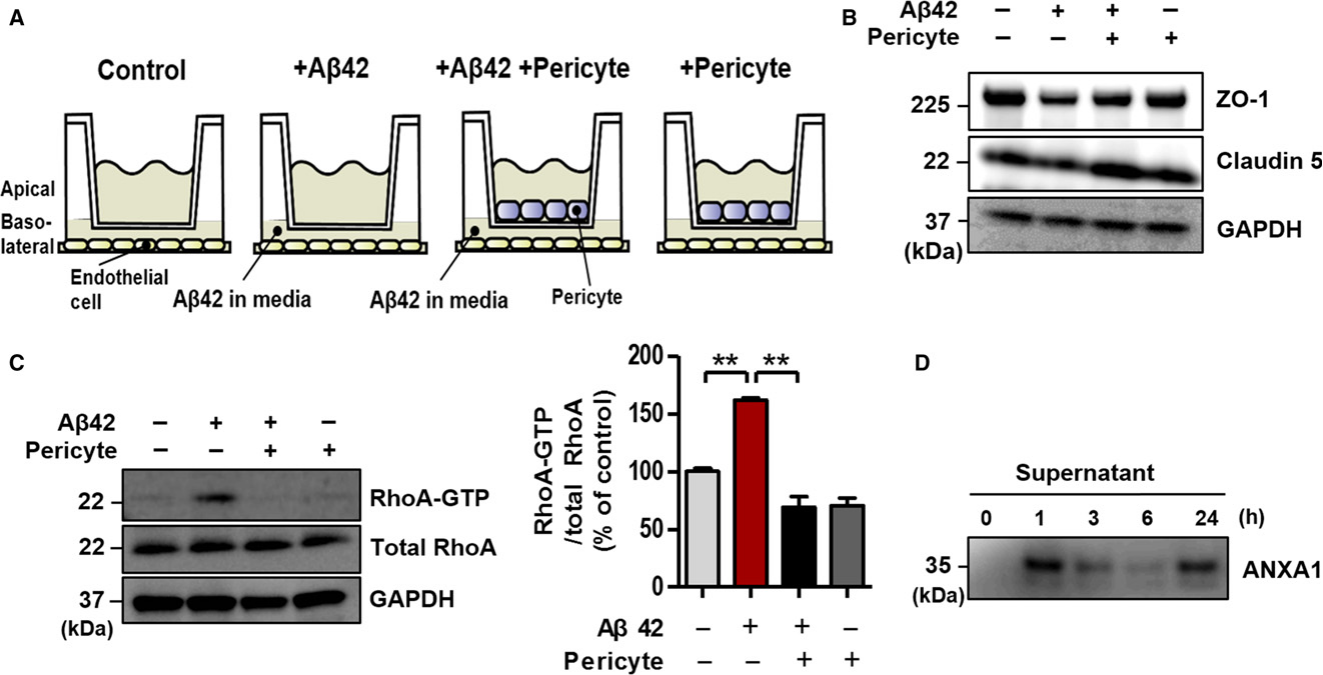
Pericytes regulate blood–brain barrier integrity by ANXA1 secretion and RhoA‐GTP inhibition. (A) Diagram of pericyte‐endothelial cell double‐layer culture for (B) and (C). Pericytes were cultured on apical layer, and bEnd.3 cells (endothelial cells) were laid on basolateral layer. For details, see Experimental procedure section. (B) The effect of pericytes on tight junction proteins (ZO‐1 and Claudin 5). (C) Aβ42 induced RhoA‐GTP was significantly decreased by pericytes (Aβ42, 5 μm for 1 h; **P < 0.01, ANOVA with Tukey's multiple‐comparison test, *n* = 3). RhoA‐GTP was detected by RhoA‐GTP pull‐down assay. RhoA‐GTP was pulled down by Rhotekin‐RBD beads, and it was captured by anti‐RhoA antibody. (D) ANXA1 secretion in pericytes. Secreted ANXA1 in conditioned media (supernatant) was measured by trichloroacetic acid precipitation and immuno‐blotted.

It has been reported that signaling between pericytes and endothelial cells is crucial for BBB maintenance (Bell *et al*., 2010). Pericyte‐endothelial cell double‐layer culture (apical layer, pericytes; basolateral layer, endothelial cells) was performed to investigate whether pericytes have a direct effect on the maintenance of the BBB. When pericytes were cultured with bEnd.3 cells, the Aβ42‐induced disruption of the tight junction was attenuated (Fig. 6A,B). Next, we examined whether the above result is related to RhoA activation and ANXA1 secretion by pericytes because there previous reports indicated that ANXA1 can be produced by various cell types (Perretti & D'Acquisto, 2009) and expressed in smooth muscle cells and pericytes (Solito *et al*., 2008). Interestingly, increased RhoA‐GTP by Aβ42 was significantly reduced by the pericyte‐endothelial cell double‐layer culture (Fig. 6C, **P < 0.01, *n* = 3). To identify the reason of inhibition of RhoA activation by pericytes, we performed trichloroacetic acid (TCA) precipitation and found that pericytes secrete ANXA1 (Fig. 6D). These data suggest that pericytes might regulate BBB integrity through inhibition of RhoA activation by their ANXA1 secretion.

## Discussion

Although there are substantial evidences of the protective role of ANXA1 on the maintenance of the BBB (McArthur *et al*., 2010; Cristante *et al*., 2013), its efficacy on the BBB breakdown in AD is unknown. To investigate the important role of ANXA1 in AD, we first measured the level of ANXA1 in the serum of patients with AD because previous reports showed that ANXA1 is reduced in the blood samples from various types of diseases such as obesity, sepsis, and multiple sclerosis (MS); interestingly, all of these are related to dysfunction of the BBB during the course of the disease (Minagar & Alexander, 2003; Fenton, 2006; Banks, 2008; Cristante *et al*., 2013; Kosicka *et al*., 2013; Tsai *et al*., 2013). Consequently, ANXA1 was significantly lower in the AD serum than in normal controls serum, and we thought that it might be related to the dysfunction of the BBB in AD (Fig. 2A). Although the exact mechanism of how ANXA1 in the serum of patients with AD is reduced is unclear, we can speculate several possibilities. One possibility is the involvement of monocytes because monocytes are blood‐derived cells and contain high level of ANXA1 in their cytoplasm and secrete it when they are activated (Perretti & D'Acquisto, 2009). As Aβ42 level is reduced in the serum of patients with AD (Lui *et al*., 2010), activation of monocytes by Aβ42 is decreased followed by reduction in the secretion of ANXA1 from monocytes in the blood. Also, it is reported that monocytes migrate into the brains of patient with AD (Hohsfield & Humpel, 2015). Thus, migration of monocytes into brain under AD condition decreases the chance to secrete ANXA1 from blood monocytes. However, further studies are needed to confirm several possibilities.

Our results demonstrate the disruption of the BBB (Fig. 1) and the reduced levels of endogenous ANXA1 and its receptor FPR2 in Aβ42‐treated bEnd.3 cells and brain capillaries from 5XFAD mice (Fig. 2B,D,E). Several reports showed that Aβ42 is one of the ligands of FPR2 (He *et al*., 2013) and the interaction between the Aβ42 and FPR2 affects ANXA1 and FPR2 stability (McArthur *et al*., 2010; He *et al*., 2013). Therefore, we speculated that ANXA1 in the 5XFAD mouse brain parenchyma might be ineffective due to the interruption of Aβ42 on FPR2 resulting in the unstable expression of endogenous ANXA1 (Fig. 3A,B) (Cristante *et al*., 2013). In addition, it is possible that FPR2 level is further reduced due to the internalization and degradation of FPR2–Aβ42 complex (Yazawa *et al*., 2001). Therefore, we proposed that endogenous ANXA1 and FPR2 expressions are influenced by Aβ42 and this makes the BBB more severely weakened.

Previous reports have suggested that ANXA1 inhibits RhoA‐GTP (active form of RhoA) in hCMEC/D3 cells (Cristante *et al*., 2013) and activation of RhoA triggers an increase in endothelial permeability (van Nieuw Amerongen *et al*., 2000). Additionally, it has been shown that inhibition of RhoA‐GTP attenuates the BBB permeability (van Nieuw Amerongen & van Hinsbergh, 2007) and RhoA is activated in the cerebral cortex of the Tg2576 mice model of AD that overexpresses a mutant form of APP (KM670/671NL) (Petratos *et al*., 2008). In addition, it has been generally known that signaling and interaction between pericytes and endothelial cells are critical for the BBB maintenance (Bell *et al*., 2010). Based on these preceding studies, we investigated whether (i) Aβ42 induces RhoA activation in endothelial cells, (ii) inhibition of RhoA restores Aβ42‐induced BBB dysfunction, (iii) ANXA1 effects are related to RhoA inhibition, and (iv) pericytes regulates the BBB integrity by secretion of ANXA1 and inhibition of RhoA activation. Consequently, our results showed that RhoA was activated in the Aβ42‐treated bEnd.3 cells and in the brain capillaries of 5XFAD mice (Fig. 4A,B). Also, Y27632 (ROCK inhibitor) and Rhosin (inhibitor of GEF‐catalyzed RhoA activation) significantly restored tight junction protein levels and integrity of bEnd.3 monolayer, which was worsened by Aβ42 treatment (Fig. 4C,D,E). Finally, bEnd.3 cells were transfected by the mutant cDNA of RhoA (Q63L, constitutively active form). When bEnd.3 cells overexpressed RhoA‐GTP, Aβ42‐induced RhoA activation was accelerated, and then, ANXA1 could not prevent Aβ42‐triggered reduction in tight junction proteins (Fig. 5). Furthermore, the fact that RhoA‐GTP alone could not decrease tight junction proteins suggests that RhoA activation specifically induced by Aβ42 is linked more closely with the BBB disruption in AD. However, to more specifically identify that the effect of ANXA1 is due to the inhibition of RhoA activation, we need additional experiments about possible mechanisms of ANXA1. There are some reports that (i) ANXA1 inhibits NMDAR activation (Black *et al*., 1992), (ii) NMDAR activation induces BBB disruption (Koenig *et al*., 1992), and (iii) Aβ42 increases NMDAR activation (Texido *et al*., 2011). According to these reports, the protective effect of ANXA1 could be caused by not only the inhibition of RhoA‐GTP but also the inhibition of NMDAR. However, when we cotreated MK801 (NMDAR antagonist) with Aβ42, it did not recover the BBB integrity which had been broken by Aβ42 (Fig. S3B, *P* > 0.05). Also, Fig. S3C (Supporting information) shows that the protective effect of ANXA1 against Aβ42 was not diminished by the treatment of NMDAR agonist (l‐glutamate). This means that the effect of ANXA1 has little to do with the activation states of NMDAR. Finally, pericytes regulate the BBB integrity (Fig. 6B) by RhoA‐GTP inhibition (Fig. 6C) and ANXA1 secretion (Fig. 6D). Taken together, our results indicate that ANXA1 rescues the BBB integrity by the specific inhibition of RhoA‐GTP and this phenomenon also can be induced by pericyte‐derived ANXA1 secretion to endothelial cells.

Collectively, the novel findings in this study are the followings: (i) ANXA1 and FPR2 are reduced in the Aβ42‐treated bEnd.3 cells and the capillaries of 5XFAD mice, (ii) ANXA1 is decreased in the serum of patients with AD, (iii) Aβ42 induced RhoA activation resulting in the disruption of the BBB, (iv) ANXA1 rescues Aβ42‐induced BBB disruption through the inhibition of RhoA‐GTP, (v) pericyte also regulates BBB integrity by secretion of ANXA1 and inhibition of RhoA‐GTP. Figure S1 (Supporting information) shows a schematic explanation of our hypothesis. Our study provides the strong evidences that ANXA1 has a critical role in maintaining the BBB integrity and proves its ability to prevent Aβ42‐induced BBB disruption by inhibition of RhoA activation. Therefore, we suggest that ANXA1 is a possible therapeutic target for managing the dysfunction of the BBB in AD.

## Experimental procedures

### Reagents

Aβ42 peptide was purchased from American Peptide Company (Sunnyvale, CA, USA) and was prepared as previously described (Byun *et al*., 2015). It was dissolved in hexafluoroisopropanol for 72 h at room temperature (RT) and lyophilized. The peptide was then dissolved again in dimethylsulfoxide. Anti‐ZO‐1, anti‐Claudin 5 (Thermo Fisher Scientific, Waltham, MA, USA), anti‐GAPDH (Abcam, Cambridge, MA, USA), anti‐GFP (Santa Cruz Biotechnology, Inc., Santa Cruz, CA, USA), anti‐CD31 (R&D Systems, Minneapolis, MN, USA), antiphospho CREB (Cell signaling Technology, Danvers, MA, USA), anti‐CREB (Cell signaling Technology), and anti‐Annexin A1 (Invitrogen, Carsbad, CA, USA) were used for Western blot analysis, and anti‐ZO‐1 (Thermo Fisher Scientific) was used for immunofluorescence images. Y27632, MK801, and l‐glutamate were purchased from Sigma‐Aldrich Co. (St. Louis, MO, USA), Rhosin was purchased from Merck Millipore (Billerica, MA, USA), and human Annexin A1 (ANXA1) recombinant protein was purchased from MyBioSource (San Diego, CA, USA). For experiments, bEnd.3 cells and/or pericytes were treated with Aβ42 (2 μm and/or 5 μm; from 0 to 24 h, differently for each experiment), ANXA1 (1 μg mL^−1^ and/or 2 μg mL^−1^; for 24.5 h), Y27632 (30 μm; for 24 h), Rhosin (10 μm; for 24 h), l‐glutamate (30 μm; for 30 min or 24 h), and MK801 (10 μm; for 30 min or 24 h).

### Animals and tissue collection

Transgenic mice with AD‐associated five familial mutations (5XFAD) were purchased from The Jackson Laboratory (Bar Harbor, ME, USA). These mice express high levels of both mutant human amyloid precursor protein (APP695) with Swedish mutation (K670N, M671L), London mutation (V717I), Florida mutation (I716V), and human presenilin 1 (PS1) with two mutations (M146L and L286V). Animal management and experimentation were carried out in strict conformity with the Principle of Laboratory Animal Care (NIH publication, revised 1985; Bethesda, Maryland, USA) and the Animal Care and Use Guidelines (Seoul National University; Seoul, Korea). In addition, we made earnest efforts to minimize animal suffering and to lessen the number of mice used.

### Cell culture

The murine brain endothelial cell line bEnd.3 (ATCC, Manassas, VA, USA) and primary human microvascular pericyte cells (from Dr. Chung‐Hyun Cho, Seoul National University, Seoul, Korea) were cultured in Dulbecco's modified Eagle's medium (DMEM; Hyclone, Irvine, CA, USA) that was supplemented with 10% fetal bovine serum (FBS; Hyclone), 100 U mL^−1^ penicillin, and 100 μg mL^−1^ streptomycin (Sigma‐Aldrich Co.) and incubated at 37 °C in 5% CO_2_ incubator. For pericyte‐endothelial cell double‐layer culture, Corning Transwell polycarbonate membrane cell culture inserts (6.5‐mm or 24‐mm transwell with 0.4 μm pore; Corning, NY, USA) were used. Pericytes were cultured on the apical layer of transwell (6.5‐mm, 6 × 10^4^ cells cm^−2^; 24‐mm, 1.8 × 10^5^ cells cm^−2^), and bEnd.3 cells were seeded on the basolateral layer of transwell (6.5‐mm, 1.2 × 10^5^ cells cm^−2^; 24‐mm, 3.6 × 10^5^ cells cm^−2^).

### Mouse brain capillary isolation

Mouse brain capillaries were isolated from the wild‐type (WT) littermates and 5XFAD mice at 9–10 months of age, as previously described (Park *et al*., 2014). Shortly, mice were euthanized at the end of the study by CO_2_ euthanasia and then decapitated. Mouse brains were gently homogenized in PBS supplemented with 5 mm D‐glucose and 1 mm sodium pyruvate (pH 7.4). The homogenate was centrifuged at 5800 *g* for 20 min at 4 °C with the addition of Ficoll (final concentration 15%). The pellets were resuspended in PBS with 1% BSA and passed over a glass bead column (0.3–0.4 mm glass beads). The capillaries adhere to the glass beads while the other impurities pass unimpeded. Capillaries were recovered and lysed by gentle agitation in radio‐immunoprecipitation assay (RIPA) buffer (150 mm NaCl, 1% sodium dodecyl sulfate, and 50 mm Tris–HCl, pH 7.4) containing protease inhibitors (Sigma‐Aldrich Co.) and phosphatase inhibitors (A.G. Scientific, Inc., San Diego, CA, USA).

### Western blot analysis

bEnd.3 cells and isolated mouse brain capillaries were lysed with RIPA buffer containing protease inhibitors and phosphatase inhibitors. Proteins were extracted and quantified by a bicinchoninic (BCA) protein assay. The lysates were equally loaded on 10% glycine gels or 4–12% Nupage bis‐tris gels (Thermo Fisher Scientific) to be separated according to size. The samples were transferred to a polyvinylidenedifluoride (PVDF) membrane for 90 min at 70 V, and the membrane was blocked with 5% skim milk in Tris‐buffered saline with 0.05% Tween 20 (TBST) for 1 h. After blocking, it was incubated with primary antibodies in TBST (with 3% BSA and 0.05% sodium azide) overnight at 4 °C, and the following day it was incubated for 1 h with secondary antibodies in TBST at RT. The protein bands on the PVDF membrane were visualized with a bio‐imaging analyzer (LAS‐3000; Fujifilm Corporation, Tokyo, Japan) with a chemiluminescence detection solution (Ab Frontier Co., Seoul, Korea). The images were analyzed with a Multi‐Gauge program (Fujifilm Corporation).

### Trichloroacetic acid protein precipitation

Trichloroacetic acid protein precipitation was carried out to measure the levels of secreted ANXA1 from pericytes. Conditioned medium from pericyte cells was incubated with trichloroacetic acid TCA solution overnight at 4 °C, centrifuged at 18 000 g at 4 °C for 5 min, and the supernatant was removed. The pellets were resuspended with 100% ice‐cold acetone, air‐dried at 95 °C for 5 min, and boiled with 25 μL of 2× sample buffer for 10 min at 95 °C. The samples were loaded on the 4–12% Nupage bis‐tris gels (Thermo Fisher Scientific) for immunoblotting.

### Immunocytochemistry

bEnd.3 cells were seeded onto a four‐well cell culture chamber slide (SPL Lifesciences, Gyeonggi‐do, Korea). After the treatment of drugs, the slide was washed twice with ice‐cold PBS and fixed with 4% paraformaldehyde (PFA) solution for 10 min at RT. After permeabilization with Triton X‐100 (0.3%) in PBS, the cells were incubated with anti‐ZO‐1 antibody (1:100) overnight at 4 °C. After washing with PBS, the cells were incubated again with fluorescent‐labeled secondary antibodies (1:500; Life Technologies, Waltham, MA, USA) for 1 h at RT and counterstained with 4′‐6‐diamidino‐2‐phenylindole (DAPI) for 10 min at RT. The images were taken by a confocal laser scanning microscope (LSM700; ZEISS international, Oberkochen, Germany) and analyzed with the Image J software (National Institutes of Health, Bethesda, MD, USA). In detail, we measured the lengths of continuous borders of TJ proteins and calculated mean connected TJ lengths in one cell, and the value was divided by total perimeter of the cell. This performance was taken four times per one image (*n* = 4, four different cells per one captured image), and we found the average ratio of connected TJ lengths per each total cell perimeter (connected TJ/cell perimeter) of one image. The lower value of connected TJ/cell perimeter means the more severe disruption of BBB. Four images (*n* = 4, independent experiments) in each group were used for quantification and comparison.

### *In vitro* BBB permeability assay

bEnd.3 cells were seeded (6 × 10^4 ^cells cm^−2^) onto the 6.5‐mm transwell with a 0.4‐μm pore polycarbonate membrane insert (Corning). The cells were incubated in DMEM supplemented with 10% FBS and 100 U mL^−1^ penicillin and 100 μg mL^−1^ streptomycin within three days of seeding. After three days, the permeability of bEnd.3 cells was measured using fluorescein isothiocyanate‐conjugated dextran (FD‐40, MW 40,000; Sigma‐Aldrich) (Kook *et al*., 2012). FD‐40 (0.1 mg mL^−1^) in opti‐MEM Media (Gibco, Waltham, MA, USA) was added to the apical compartment and the media in the basolateral compartment was replaced by opti‐MEM. After 30 min at 37 °C in 5% CO_2_ incubator, the samples collected from the basolateral compartment were moved onto a 96‐well plate (Corning). The fluorescence of samples (A.U.) was measured on a fluorescence luminometer (Tecan Systems Inc., Seestrasse, Mannedorf, Switzerland) at the wavelengths of 490 nm excitation and 520 nm emission.

### RNA isolation and Real‐time PCR

To measure mRNA levels in the bEnd.3 cells, RNA isolation and quantitative real‐time PCR were performed. Briefly, total RNA was extracted from bEnd.3 cells using the Qiagen RNeasy kit (Qiagen, Valencia, CA, USA). cDNA was synthesized from mRNA using Maxime RT Premix Oligo dT primer kit (Intron Biotechnology, Seoul, Korea). ANXA1 primer (forward primer: 5′‐GAAACCATCTGAGCAGAGTCTCTC‐3′, reverse primer: 5′‐CATCCGAGGATACATTGAAGGAAG‐3′) and GAPDH primer (forward primer: 5′‐AATGTGTCCGTCGTGGATCT‐ 3′, reverse primer: 5′‐GGTCCTCAGTGTAGCCCAAG‐3′) were used. Real‐time PCR was carried out using the ABI Step One 2.1 (Applied Biosystems, Foster City, CA, USA). GAPDH was used as an endogenous control for real‐time PCR.

### Transfection

Transfection was performed by Lipofectamine LTX according to the manufacturer's instructions (Invitrogen). The pcDNA3 vector was used as a control to normalize the efficiency of transfection, and pcDNA3‐EGFP‐RhoA‐Q63L (Q63L, mutant of RhoA constitutively active; Addgene, Cambridge, MA, USA) was used for RhoA‐GTP overexpression. The cDNA constructs were mixed with Lipofectamine Plus and Lipofectamine LTX (Invitrogen) in opti‐MEM. After 20 min of incubation, the mixture was centrifuged and added to the culture medium of bEnd.3 cells for 24 h, and then, the cells were treated with the specific drugs.

### Enzyme‐linked immunosorbent assay (ELISA) using human blood serum samples

To determine the concentrations of human serum ANXA1, the human ANXA1 ELISA Kit (Cusabio, Hubei, China) was used in accordance with the manufacturer's guidelines. In brief, prepared serum samples and standards were added to each well and incubated for 2 h at 37 °C. Biotin antibody was added, and the samples were incubated for 1 h at 37 °C. After washing, horseradish peroxidase (HRP)‐avidin was added to be conjugated to biotin antibody and incubated for 1 h at 37 °C, and then, TMB substrate was used for detection of HRP activity. The plate was read at 450 nm within 5 min.

### Demographic data of subjects

Twenty cognitively normal control subjects (sex ratio, M:F = 7:13; average age ± SEM, 66.9 ± 1.0) and 14 participants with AD (sex ratio, M:F = 4:10; average age ± SEM, 71.1 ± 2.5) were included in this study. There were no significant intergroup differences for sex (*P* > 0.05, *chi*‐square test, χ^2^ = 0.151) and age (*P* > 0.05, unpaired *t*‐test) (see the Table S1). Human serum samples were obtained from Dr. Jong‐Won Kim at Samsung Medical Center (SMC) in Korea, and this work was approved by Institutional Review Board (IRB) in Korea.

### Active RhoA pull‐down assay

To measure the level of RhoA‐GTP (GTP‐bound state, active form of RhoA) in the bEnd.3 cells, mouse brain capillaries, and capillary‐depleted brain samples, RhoA activation assay biochem kit (Cytoskeleton, Denver, CO, USA) was used in accordance with the manufacturer's guidelines. In brief, the samples were lysed with cell lysis buffer (RIPA buffer) and incubated with predetermined amount of Rhotekin‐RBD beads (bind specifically to the GTP‐bound form of Rho) for 1 h at 4 °C. Next, the samples were centrifuged at 5000 *g* at 4 °C for 1 min, and the bead pellets were washed with wash buffer. After centrifugation, again the supernatant was removed, and remaining bead pellets were boiled with 10 μL of 2× laemmli sample buffer for 5 min at 85 °C. The samples were loaded on the 4–12% Nupage bis‐tris gels (Thermo Fisher Scientific). Anti‐RhoA antibody was used for immunoblotting.

### Immunohistochemistry

Immunohistochemistry for IgG staining was performed as described previously with some modifications. Briefly, 9‐ to 10‐month‐old mice were anesthetized and perfused sequentially with PBS and 4% PFA in PBS, and the brain samples were fixed again in 4% PFA for 20 h. The slices were sectioned with a CM 1950 cryostat (Leica Microsystems GmbH, Nussloch, Germany). For IgG staining, the mouse brain sections were washed twice with ice‐cold PBS and pretreated with 3% H_2_O_2_ for 30 min at room temperature. After washing three times, the sections were incubated overnight with biotinylated anti‐mouse IgG antibody (Vector, Burlingame, CA, USA) and then visualized with 3,3‐diaminobenzidine tetrahydrochloride (DAB) by the avidin–biotin–peroxidase complex (ABC) method. Tissues were mounted, air‐dried, dehydrated by alcohol, and immersed in xylene. They were cover slipped with Permount solution (Thermo Fisher Scientific) and imaged using a fluorescence microscope (IX71; Olympus Corporation, Tokyo, Japan). The acquired images (prefrontal cortex regions) were analyzed by Image J software. IgG intensity was measured under the same threshold line, and the average values were determined and quantified.

### *In vivo* measurement of BBB permeability

An *in vivo* BBB permeability test was measured using sodium fluorescein (NaFI, 0.376 kDa) as previously described with minor modifications (Shimojima *et al*., 2008; Chen *et al*., 2013). Mice were administered 0.1 mL of NaFI (0.1 g mL^−1^) by intraperitoneal (i.p.) injection and after 45 min they were anesthetized with a mixture of Zoletil 50 (Virbac, Carros, France) and Rompun (Bayer Korea, Seoul, Korea). Five minutes later, mice were perfused with 50 mL 1× PBS and then decapitated. The brains were homogenized in 30% TCA solution and immediately centrifuged at 10 000 *g* for 5 min. NaFI concentration of the supernatant was measured by a fluorescence luminometer (Tecan Systems Inc.) at excitation (460 nm) and emission (515 nm).

### MTT assay

To check cell viability, we conducted MTT (3‐(4,5‐dimethylthiazol‐2‐yl)‐2,5‐diphenyltetrazolium bromide) assay for bEnd.3 cells (Son *et al*., 2014). Briefly, after drug treatment, 2.5 mg mL^−1^ of MTT (Sigma‐Aldrich) in opti‐MEM media was added and incubated for 1 h at 37 °C. After dissolution formazan crystals with isopropanol for 2 h, absorbance was measured at 540 nm. Experiments were repeated eight times (*n* = 8), independently.

### Statistics

Statistical analysis was carried out using GraphPad Prism 5 (GraphPad software, San Diego, CA, USA). All data were displayed as mean ± SEM and analyzed by unpaired *t*‐test (to compare exactly two groups) or one‐way ANOVA with Tukey's multiple‐comparison *post hoc* test (to compare three groups or more). In addition, we conducted Pearson's chi‐square test (χ^2^) to compare the two intragroup variables using Medcalc (Medcalc software, Ostend, Belgium). Statistically significant results were shown as appropriate *P*‐value (*P < 0.05, **P < 0.01, ***P < 0.001; symbols were described in the order of *, †, ‡, §, and #).

## Author Contributions

JC Park, SH Baik, SH Han, HJ Cho, and IH Mook‐Jung designed the study. JC Park, SH Baik, HJ Cho, HJ Choi, HJ Kim, HS Choi, WI Lee, and DK Kim performed the experiments. JC Park interpreted the data and wrote the manuscript. IH Mook‐Jung examined the earlier draft of the manuscript in detail and edited it. JC Park managed and analyzed all other data. JC Park had full access to all of the data in the study.

## Conflict of Interest

None declared.

## Funding

No funding information provided.

## Supporting information


**Fig. S1** An illustration of the hypothesis resulting from our experiments.
**Fig. S2** MTT assay for Aβ42 treated bEnd.3 cells.
**Fig. S3** The effect of ANXA1 is not related with NMDAR activation.

**Table S1** Demographic data of subjects.
**Table S2** Antibodies.
