# Supplementary material for: Annexin A1 restores Aβ1‐42‐induced blood–brain barrier disruption through the inhibition of RhoA‐ROCK signaling pathway
Source: Aging Cell. 2016 Sep 16;16(1):149–61. doi: 10.1111/acel.12530 (PMC5242298; doi:10.1111/acel.12530)
Supplement: Supplementary file 1 — Fig. S1 An illustration of the hypothesis resulting from our experiments. Fig. S2 MTT assay for Aβ42 treated bEnd.3 cells. Fig. S3 The effect of ANXA1 is not related with NMDAR activation. [file ACEL-16-149-s001.docx]

**Supporting Information**

**
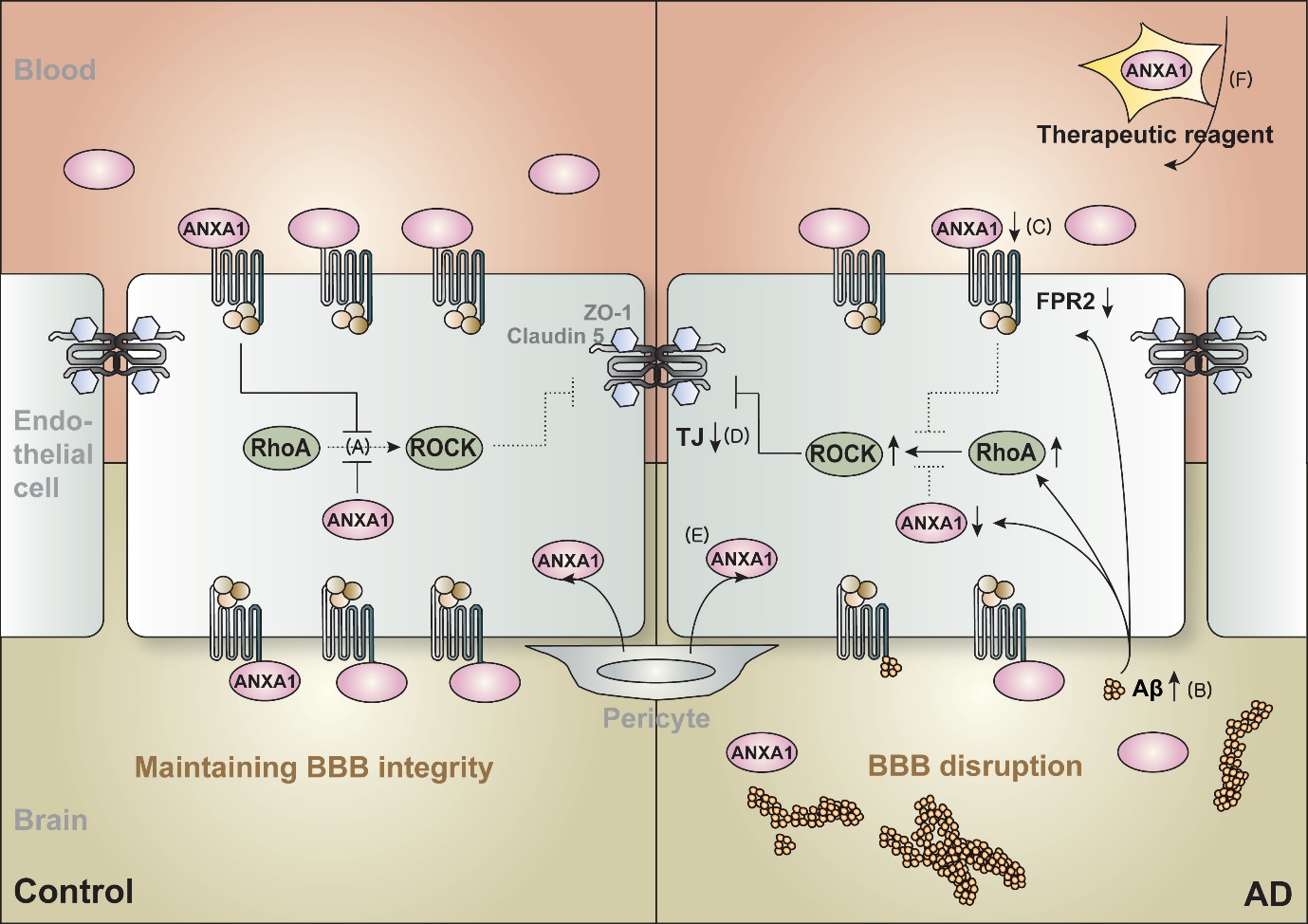
**

**Figure S1. An illustration of the hypothesis resulting from our experiments** (A) ANXA1 has a protective role in maintaining the BBB integrity in controls by blocking RhoA activation. (B) Aβ42 induces RhoA activation, decrease in endogenous ANXA1, and reduction of endothelial FPR2 receptors. (C) The level of serum ANXA1 was diminished in AD. (D) Aβ42 lessens the length of tight junction (TJ) and the number of TJ proteins. (E) Pericytes help maintain the integrity of the BBB by producing ANXA1. (F) Exogenously treated ANXA1 has protective effects against BBB disruption in AD.

**Figure S2. MTT assay for Aβ42 treated bEnd.3 cells** Aβ42 (5 μM for 24 h) did not significantly decrease the cell viability (P > 0.05 versus control, unpaired *t*-test; N = 8).


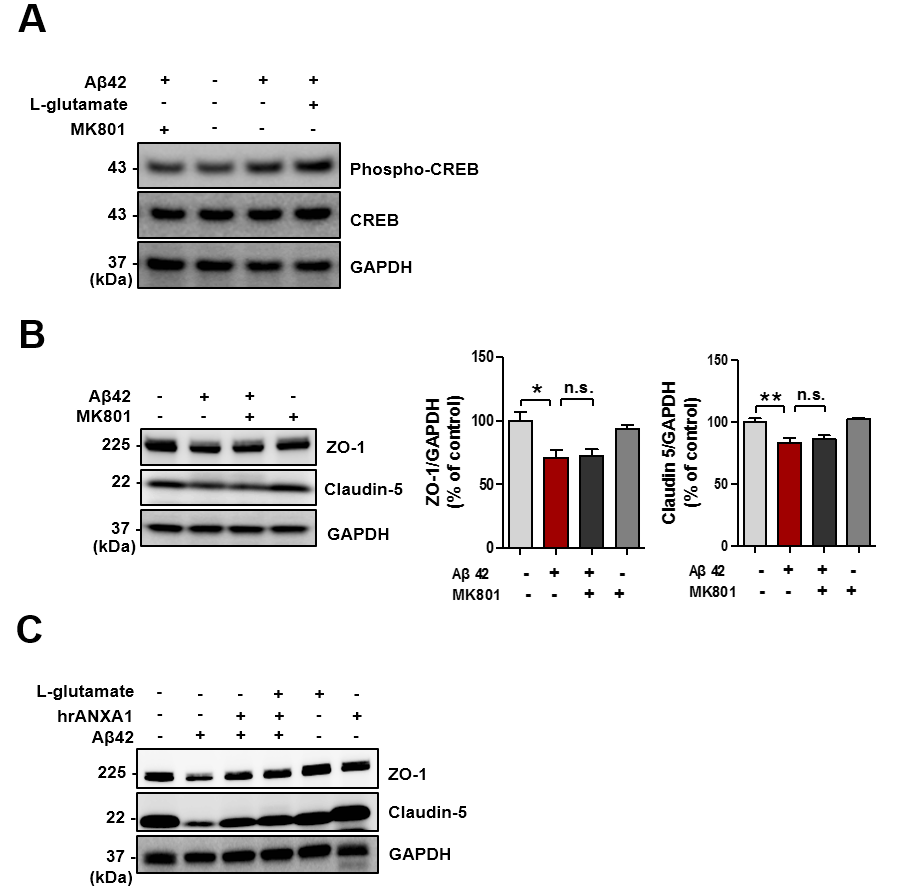


**Figure S3. The effect of ANXA1 is not related with the NMDAR activation** (A) Experimental validation of appropriate MK801 (NMDAR antagonist, 10 μM for 30 min) and L-glutamate (NMDAR agonist, 30 μM for 30 min) treatment concentration condition. Phospho-CREB was used as a positive control reflecting NMDAR activation states. (B) MK801 did not rescue the Aβ42 induced reduction of tight junction proteins (Aβ42, 5 μM for 24 h; MK801, 10 μM for 24 h; P > 0.05, ANOVA with Tukey’s multiple-comparison test; N = 4). (C) Protective effect of ANXA1 was not influenced by the activation of NMDAR (Aβ42, 5 μM for 24 h; hrANXA1, 1 μg/ml, pre-treated for 30 min before Aβ42 treatment and incubated with Aβ42 for 24 h; L-glutamate, 30 μM for 24 h).
