# Supplementary material for: Annexin A1 restores Aβ1‐42‐induced blood–brain barrier disruption through the inhibition of RhoA‐ROCK signaling pathway
Source: Aging Cell. 2016 Sep 16;16(1):149–61. doi: 10.1111/acel.12530 (PMC5242298; doi:10.1111/acel.12530)
Supplement: Supplementary file 2 — Table S1 Demographic data of subjects. Table S2 Antibodies. [file ACEL-16-149-s002.docx]

|  | Control (N = 20) | AD (N = 14) | Significant level (^a^P) |
| --- | --- | --- | --- |
| Age ± SEM (year) | 66.9 ± 1.0 | 71.1 ± 2.5 | 0.0812 |
| Gender (M/F) | 7/13 | 4/10 | 0.6976 |
| ANXA1 ± SEM (ng/mL) | 1.8 ± 0.2 | 1.2 ± 0.1 | 0.0096 |

**Table S1. Demographic data of subjects**

**-** ^a^**P value by unpaired *t*-test for age and ANXA1, and *chi-*square test (χ^2^ = 0.151) for gender**

**- SEM, Standard error of mean; ANXA1, Annexin A1**

**Table S2. Antibodies**

| Antibody | Cat.No | Supplier | Clonality &Isotype | Dilution | Host | Method |
| --- | --- | --- | --- | --- | --- | --- |
| Anti- ZO1 | 61-7300 | ThermoFisher  Scientific | Polyclonal  /IgG | 1:250  1:1000 | Rabbit | ICC  WB |
| Anti-Claudin 5 | 34-1600 | ThermoFisher  Scientific | Polyclonal  /IgG | 1:1000 | Rabbit | WB |
| Anti- Claudin 5 | 352588 | ThermoFisher  Scientific | Monoclonal  /IgG1 | 1:250 | Mouse | ICC |
| Anti-GAPDH | ab9485 | Abcam | Polyclonal  /IgG | 1:2000 | Rabbit | WB |
| Anti- IgG | BA-2000 | Vector  Laboratories | Biotinylated  Secondary Antibody | 1:400 | - | IHC |
| Anti-ANXA1 | 71-3400 | ThermoFisher  Scientific | Polyclonal  /IgG | 1:1000 | Rabbit | WB |
| Anti- FPR2 | NLS1878 | Novus  Biologicals | Polyclonal  /IgG | 1:750 | Rabbit | WB |
| Anti-β tubulin | T8535 | Sigma  Aldrich | Monoclonal  /IgG2b | 1:2000 | Mouse | WB |
| Anti- CD31 | AF3628 | R&D  Systems | Polyclonal  /IgG | 1:400 | Goat | WB |
| Anti- 6E10 | SIG-39320-200 | Covance  From  Biolegend | Monoclonal  /IgG1 | 1:1000 | Mouse | WB |
| Anti- RhoA | ARH03 | Cytoskeleton | Monoclonal  /IgG | 1:500 | Mouse | WB |
| Anti- GFP | sc9996 | Santa Cruz  Biotech | Monoclonal  /IgG2a | 1:500 | Mouse | WB |
| Anti-p-CREB | #9198s | Cell signaling  Technology | Monoclonal  /IgG | 1:1000 | Rabbit | WB |
| Anti- CREB | #9197s | Cell signaling  Technology | Monoclonal  /IgG | 1:1000 | Rabbit | WB |
